# Prioritization of Copy Number Variation Loci Associated with Autism from AutDB–An Integrative Multi-Study Genetic Database

**DOI:** 10.1371/journal.pone.0066707

**Published:** 2013-06-18

**Authors:** Idan Menashe, Eric C. Larsen, Sharmila Banerjee-Basu

**Affiliations:** 1 MindSpec, McLean, Virginia, United States of America; 2 Department of Public Health, Faculty of Health Sciences, Ben Gurion University of the Negev, Beer-Sheva, Israel; Harvard Medical School, United States of America

## Abstract

Copy number variants (CNVs) are thought to play an important role in the predisposition to autism spectrum disorder (ASD). However, their relatively low frequency and widespread genomic distribution complicates their accurate characterization and utilization for clinical genetics purposes. Here we present a comprehensive analysis of multi-study, genome-wide CNV data from AutDB (http://mindspec.org/autdb.html), a genetic database that accommodates detailed annotations of published scientific reports of CNVs identified in ASD individuals. Overall, we evaluated 4,926 CNVs in 2,373 ASD subjects from 48 scientific reports, encompassing ∼2.12×10^9^ bp of genomic data. Remarkable variation was seen in CNV size, with duplications being significantly larger than deletions, (*P*  =  3×10^−105^; Wilcoxon rank sum test). Examination of the CNV burden across the genome revealed 11 loci with a significant excess of CNVs among ASD subjects (*P*<7×10^−7^). Altogether, these loci covered 15,610 kb of the genome and contained 166 genes. Remarkable variation was seen both in locus size (20 - 4950 kb), and gene content, with seven multigenic (≥3 genes) and four monogenic loci. CNV data from control populations was used to further refine the boundaries of these ASD susceptibility loci. Interestingly, our analysis indicates that 15q11.2-13.3, a genomic region prone to chromosomal rearrangements of various sizes, contains three distinct ASD susceptibility CNV loci that vary in their genomic boundaries, CNV types, inheritance patterns, and overlap with CNVs from control populations. In summary, our analysis of AutDB CNV data provides valuable insights into the genomic characteristics of ASD susceptibility CNV loci and could therefore be utilized in various clinical settings and facilitate future genetic research of this disorder.

## Introduction

Copy number variations (CNVs) are structural chromosomal aberrations, which are giving rise to gains or losses of certain genomic loci across the human genome [1], [2]. While most CNVs have no apparent phenotypic consequences, there is increasing evidence that a number of chromosomal micro deletions or duplications at specific locations are involved in the predisposition of various human diseases [3], [4]. Recent advances in high-resolution, high-throughput genomics technologies have facilitated the detection of CNVs in large-scale genetic studies. Moreover, the continuous drop in labor and cost associated with these technologies promote their inclusion in genetic screening for pre- and post- pregnancy tests.

Autism spectrum disorder (ASD) constitutes a collection of clinically heterogeneous disorders that are characterized by impairments in social interactions, deficits in language and communication, and increased repetitive or stereotypic movements [5], [6]. ASD is highly heritable with estimates ranging between 40-90% heritability [7], [8]. However, given the genetically heterogeneous nature of ASD, the underlying genetic mechanisms of these disorders remain vague. Recent genetic studies have indicated that rare CNVs may play an important role in ASD susceptibility [9]–[14], and today they are considered one of the common genetic contributors of ASD [15]. Given the evidence that CNVs are a significant genetic risk factor not only for ASD, but for other developmental deficits and congenital anomalies, it has recently been proposed that chromosomal microarray (CMA) screening replace conventional cytogenetic techniques as a first-tier clinical diagnostic test for individuals with these disorders [16], [17]. However, the relatively low frequency and widespread genomic distribution of these variants in ASD cases complicates the clinical utilization of CMA screening as a potential diagnostic tool.

Given the important role of CNVs in ASD genetics and the increasing usage of CMA screening for the genetic evaluation of ASD individuals, there is a tremendous need to consolidate the large amounts of CNV data that were generated from ASD subjects and subsequently prioritize the most consistent genomic loci associated with ASD susceptibility. To this end, we explored the CNV data available at the online autism genetic database AutDB (http://www.mindspec.org/autdb.html) which, to the best of our knowledge, is the most comprehensive online resource of curated genetic data of ASD from published scientific reports. Using a range of statistical and bioinformatics analyses we performed a comprehensive and rigorous assessment of CNVs that were observed in ASD cohorts across multiple published reports consisting of both large-scale whole-genome studies and smaller-scale case studies. We subsequently determined the genomic boundaries and genetic characteristics of 11 loci demonstrating significant CNV burden among ASD subjects.

## Materials and Methods

### CNV data

For this study, we used CNV data available at the CNV module of the AutDB database (data freeze of October 2011)[18]. As with the other modules of AutDB, content of the CNV module originates entirely from published, peer-reviewed scientific literature and is rigorously annotated by scientists. Preliminary screening of reports for inclusion in the database resulted from a search of the scientific literature using PubMed (http://www.ncbi.nlm.nih.gov/pubmed/) with the following keywords: “autism/autistic/ASD” and “copy number/CNV/deletion/duplication/chromosome/structural variant”. Furthermore, CNV reports listed in ASD review articles that were not identified in the initial PubMed search were included for consideration. Next, the initial candidate CNV reports were filtered to remove those reports that did not contain at least one ASD individual in which one or more CNVS were identified. This restriction has since been relaxed to include reports describing patients with other neurodevelopmental or neuropsychiatric disorders, such as mental retardation/intellectual disability and developmental delay; in some cases, these patients also display ASD traits, but no formal diagnosis of ASD. Detailed information on ASD and control subjects from the studied cohorts was extracted from each selected CNV report for inclusion in the CNV module database.

For the purpose of CNV prioritization we aimed at analyzing a homogeneous subset of the CNV data set from AutDB by using several filtering criteria (number of CNVs removed from the data are in square brackets): only ASD cases were used [12,416 CNVs]; patients with a disease other than ASD, such as schizophrenia, developmental delay, etc. were excluded [74 CNVs]; a genome-wide CNV discovery method (array-CGH, SNP array, or solid phase hybridization) was required for inclusion; CNVs discovered by a targeted discovery method such as FISH or qPCR were excluded [215 CNVs]; we manually screened individuals to ensure minimal overlap of patients; in some cases a given CNV report may include individuals that had previously been described in another report [104 CNVs]. In such cases, we used patient ID information included in the scientific report to identify duplicate patient entries in the CNV module dataset and subsequently use the CNV data for a given individual that contained the largest number of CNV loci [104 CNVs]. Furthermore, we restricted our analysis to CNVs with defined start and end points [218 CNVs]. Finally, to maintain a uniform CNV/individual ratio in our data, we used CNVs identified in a control cohort of unaffected matched siblings as a filter to generate a set of “case-specific” CNVs in the accompanying case cohort of the Sanders *et. al* study [14] [13,036 CNVs]. This filtering process resulted in a homogeneous data set of 4,926 CNVs in 2,373 ASD subjects gathered from 48 scientific reports.

### CNV burden

To search for genomic loci demonstrating excess of CNVs among ASD subjects, we divided the genome into consecutively distributed regions of 10 kb, and evaluated the CNV burden in each region as follows: 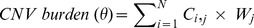
(1)


where *C_i,j_*  =  1 if a CNV (*i*) from a particular study (*j*) of all CNVs (N) in the data, overlaps with the 10 kb genomic region, and *W_,j_* is the weight associated with the study (*j*) calculated as the Loci/CNVs ratio in the study.

To assess the statistical significance of θ, we first randomly distributed the CNVs in our dataset in the human genome and then calculated their corresponding 10 kb θs. We repeated this procedure 10,000 times to generate a null genome-wide distribution of θs that fitted a Poisson distribution with λ  =  0.8363. We then used this Poisson distribution to calculate the statistical significance of θ associated with each 10 kb region among individual with ASD in our data. Given the median CNV length in our data was ∼ 43 kb, the maximal number of non-overlapping CNV loci in the human genome is ∼ 7×10^4^. Hence, we used this number to set a Bonferroni corrected cutoff for genome-wide significance of *P*<7×10^−7^ (0.05/7×10^4^). All analyses were performed using a commercial software package (MATLAB R2011b, The MathWorks Inc., Natick, MA, 2000).

### CNV loci characterization

We used the RefSeq genes track in the UCSC Genome Browser on Human Mar. 2006 (NCBI36/hg18) assembly, (http://genome.ucsc.edu) to locate genes overlapping with ASD susceptibility CNV loci. Further, we used the December 2012 release of the Human Gene module of AutDB [18] to identify genes that have been reported as containing potential susceptibility variants for ASD.

### Analysis of control CNV data

The majority of CNV loci curated in the CNV module of AutDB and used in our analysis were curated from published scientific reports in which one or more filtering steps were used to remove variants previously identified in unaffected individuals within the general population, resulting in a population of case-specific or case-enriched CNVs that were subsequently annotated. However, we concluded that an independent analysis of control CNV datasets using our prioritization strategy could be useful both in more accurately defining the boundaries of the eleven susceptibility loci identified in our initial analysis, as well as in allowing us to differentiate between potential false-positives or polymorphic CNV regions that would likely confer decreased risk of ASD susceptibility than loci with little or no control CNV overlap. Therefore, we examined the overlap of these 11 ASD susceptibility loci with CNVs among 4400 individuals with no diagnosis of ASD (controls) using data from three genome-wide CNV analyses varying in their sample sizes, studied cohorts, and CNV detection method/platform [19]–[21]. This control CNV data were collected from the Database of Genomic Variants (DGV) [22], and were analyzed using the same 10 kb regions described above.

## Results

### The CNV module of AutDB

The CNV module of AutDB as of October 2011 consisted of CNV data from 72 annotated publications, encompassing 30,989 CNVs from 4,359 individuals (3,099 ASD cases, 66 cases of other neurodevelopmental or neuropsychiatric disorders, and 1,194 control individuals). These have been summarized into 2,429 unique CNV reports classified as 'major' (i.e. independently validated) or ‘minor' (1,047 and 1,382 CNV reports respectively), and distributed across 1035 CNV loci genome-wide (Figure S1). The median number of reports per CNV locus was two (one ‘major' and one ‘minor'), with the highest number of reports per locus reaching 16 (14 and 2 'major' and 'minor' respectively) for the 16p11.2 locus (Figure S1).

### CNV dataset

For the purpose of CNV prioritization, we applied a set of stringent filtering criteria (see methods) to the AutDB data to generate a uniform subset of CNVs. This resulted in a dataset containing 4,926 CNVs in 2,373 ASD subjects (Mean  =  2.08, STD  =  1.79 CNVs per individual) collected from 48 scientific reports (Table S1) that encompassed ∼2.13×10^9^ bp of genomic data. The maximal number of CNVs per individuals seen in our data was 18. Of the 2,373 ASD subjects in our data, 1,532 were males and 330 females, which is consistent with the 4:1 reported male-to-female ratio of ASD prevalence in the general population [23]. Notably, the gender of 511 subjects in our data was not reported. Of the 4,926 CNVs in our data, 1,923 (39%) were duplications and 3,003 (61%) were deletions (Table 1). In addition, 3,377 (68.6%) of the CNVs were inherited, 239 (4.9%) were *de novo*, and 1,310 (26.6%) had no indicated inheritance (Table 1). We observed remarkable variation in CNV size and inheritance patterns, with duplications being significantly larger than deletions (*P* = 3×10^−105^; Wilcoxon rank sum test; Figure S2), and *de novo* CNVs tended to be more prevalent among females than males (*Χ^2^* = 18.6; *P*<0.0001).

**Table 1:** CNV characteristics.

	Male			Female		
	Duplications	Deletions	sum	Duplications	Deletions	sum
**De novo**	62 (1.7%)	109 (3.1%)	171 (4.8%)	27 (3.5%)	40 (5.1%)	67 (8.9%)
**Inherited**	1046 (29.5%)	1663 (46.9%)	2709 (76.4%)	200 (26.5%)	343 (45.7%)	543 (71.0%)
**NR** *	188 (5.3%)	479 (13.5%)	667 (18.8%)	32 (4.0%)	122 (15.2%)	154 (20.1%)
**Sum**	1296 (36.5%)	2251 (63.5)	3547 (100.0%)	259 (34.0%)	505 (66.0%)	764(100.0%)

	NR*			All		
	Duplications	Deletions	sum	Duplications	Deletions	sum
**De novo**	1 (0.2%)	0 (0.0%)	1 (0.2%)	90 (1.8%)	149 (3.0%)	239 (4.9%)
**Inherited**	50 (8.1%)	75 (12.2%)	125 (20.3%)	1296 (26.3%)	2081 (42.3%)	3377 (68.6%)
**NR** *	317 (51.5%)	172 (28.0%)	489 (79.5%)	537 (10.9%)	773 (15.7%)	1310 (26.6%)
**Sum**	368 (59.8%)	247 (40.2%)	615 (100.0%)	1923 (39.0%)	3003 (61.0%)	4926 (100.0%)

* NR  =  Not reported.

### CNV characterization

**Figure 1:**
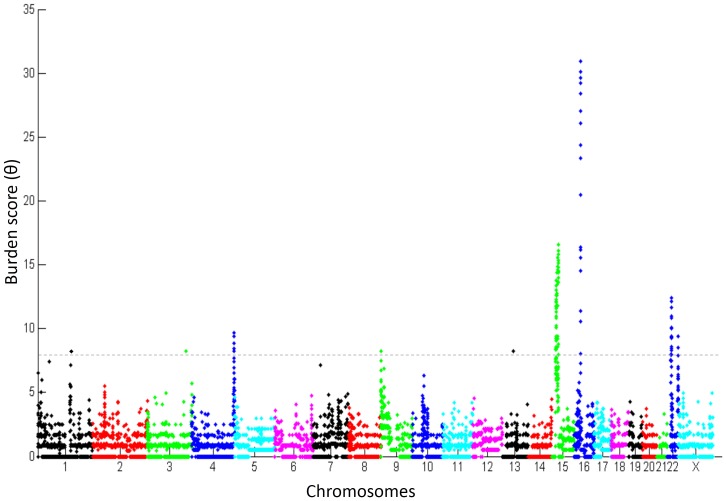
Whole-genome distribution of CNV burden. A Manhattan plot showing CNV burden among ASD subjects in 10 kb regions continuously distributed across the human genome. A dashed horizontal line indicate the burden score of 6.5 (0.995 quantile) that was used a threshold to determine the top ranked ASD susceptibility CNV loci.

**Figure 2:**
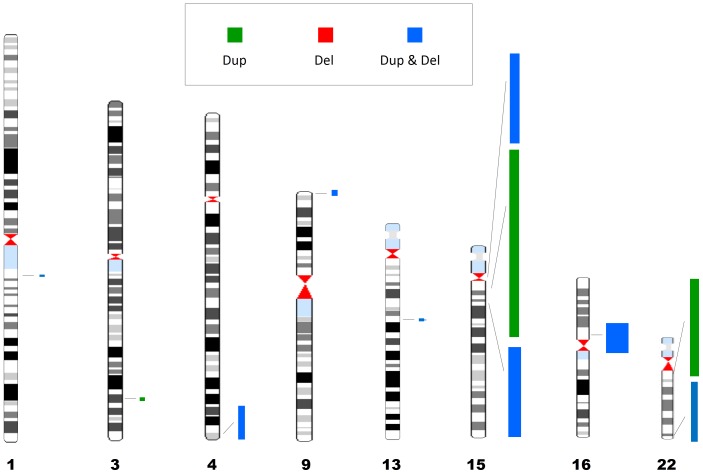
Physical locations of the top ranked 11 ASD susceptibility CNV loci on Human G-banded ideogram. CNV loci length and width are proportional to their genomic size and burden score respectively. Green, red, and blue are for CNV loci containing primarily copy number gains (Duplications), copy number losses (Deletions), or both Duplications and Deletions, respectively.

To identify genomic loci with an excess of CNVs among ASD subjects, we divided the genome into consecutively distributed regions of 10 kb and assessed the burden of CNVs within them (See Materials and Methods). The genomic distribution of CNV burden among individuals with ASD is depicted in Figure 1. Overall, there were eleven genomic loci displaying significant burden score (*P*<7×10^−7^) distributing along eight chromosomes (Figure 2; Table 2), and containing 166 RefSeq genes [24]. Of these, four loci contained only one gene (*BCL9*, *NLGN1, DOCK8*, and *KPNA3* in 1q21.1, 3q26.31, 9p24.3, and 13q14.3, respectively), one locus contained three genes (*TRIML1, TRIML2,* and *LOC401164*, on 4q35.2) and six loci contained ≥ seven genes (Table 2). Seven of these loci included a relatively equal number of duplications and deletions, whereas four loci contained a majority (≥75%) of duplications.

**Table 2:** Top ASD susceptibility CNV loci.

Genomic locus						CNVs									
Locus	Start^a^ (kb) (hg18)	End^a^ (kb)(hg18)	Size (kb)	RefSeq Genes^b^	Duplications^c^			Deletions^c^			Total	# Studies	Θ^d^	P-value^e^	
					Inh	DN	NR	Inh	DN	NR					# CNVs among controls^f^
1q21.1	145555	145575	20	BCL9	3	3	3	-	-	4	13	4	8.15	2.61×10^−7^	0
3q26.31	174745	174775	30	NLGN1	1	-	114	-	-	-	115	2	8.23	2.61×10^−7^	31
4q35.2	189295	190195	900	TRIML1TRIML2LOC401164	1	-	8	1	1	5	15	5	9.38	2.16×10^−8^	4
9p24.3	235	375	140	DOCK8	4	1	4	-	2	1	12	5	8.23	2.61×10^−7^	0
13q14.3	49265	49275	10	KPNA3	4	-	-	6	-	-	10	1	8.22	2.61×10^−7^	0
15q11.2	18525	20845	2320	9	5	6	1	6	1	-	19	7	13.38	4.31×10^−13^	384
15q11.2-q13.1	21185	26135	4950	28	3	7	20	1	-	1	32	8	13.01	4.31×10^−13^	159
15q13.2-q13.3	27985	30425	2440	7	4	8	2	8	1	3	26	9	16.60	1.11×10^−16^	272
16p11.2	29495	30245	750	31	13	5	9	2	21	13	62	10	30.96	< 1×10^−20^	0
22q11.21	17265	19805	2540	51	8	3	10	-	1	-	22	7	12.15	7.24×10^−12^	46
22q13.32-q13.33	47925	49435	1510	33	3	-	-	4	3	2	12	6	9.41	2.16×10^−8^	0
Sum			15610	167	59	32	173	45	31	34	374				
					264			110							

a The boundaries of the ASD susceptibility CNV loci were determined as the midpoint of the 10kb region with a θ>6.5.

b RefSeq genes including both protein-coding and non-coding RNA genes, and excluding Pseudogenes. For loci with >3 genes, only the number of RefSeq genes is given.

c CNV type. Inh  =  Inherited, DN  =  De-Novo, NR  =  Not Reported.

d CNV burden score.

e P-value calculated based on a Poisson distribution of CNV burden scores.

f 4400 individuals with no ASD diagnosis from three large genome-wide studies.

**Figure 3:**
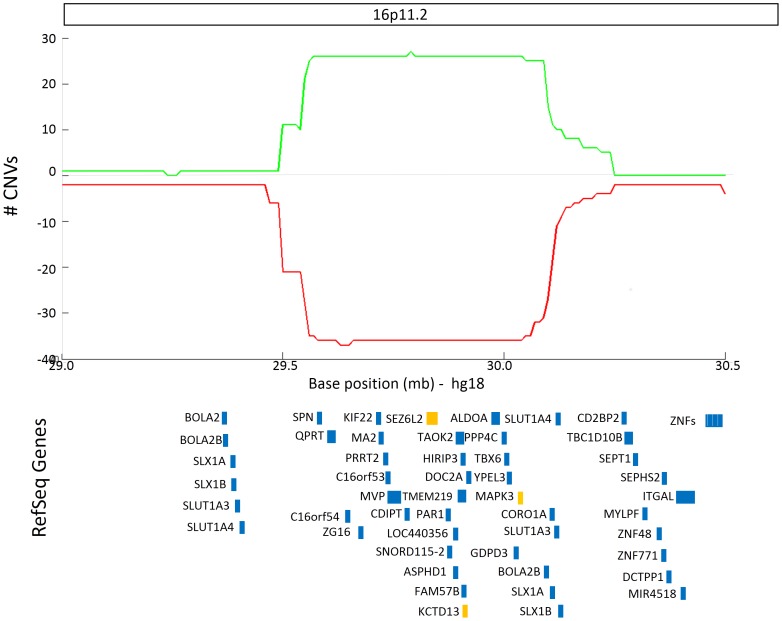
ASD susceptibility CNV locus on human chromosome 16. The number of individuals with duplications (green) and deletions (red) are plotted along human chromosome 16p11.2. RefSeq Genes overlapping with this region are depicted in blue rectangles. Genes that have been associated with ASD according to AutDB [18] are colored in orange.

The highest CNV burden was seen in a locus on human chromosome 16p11.2 (θ = 30.96; *P* <1×10^−20^), with 27 duplications and 36 deletions identified in ASD subjects from 10 different studies (Figure 3). No CNVs within this region were observed in 4400 controls. This locus spanned 750 kb and contained 31 RefSeq genes, three of which (*SEZ6L2*, MAPK3, and *KCTD13*) have been reported as containing susceptibility genetic variants in ASD individuals [25]–[27]. Interestingly, while duplications in the 16p11.2 locus tended to be inherited, deletions were overwhelmingly *de novo* in origin (Table 2).

**Figure 4:**
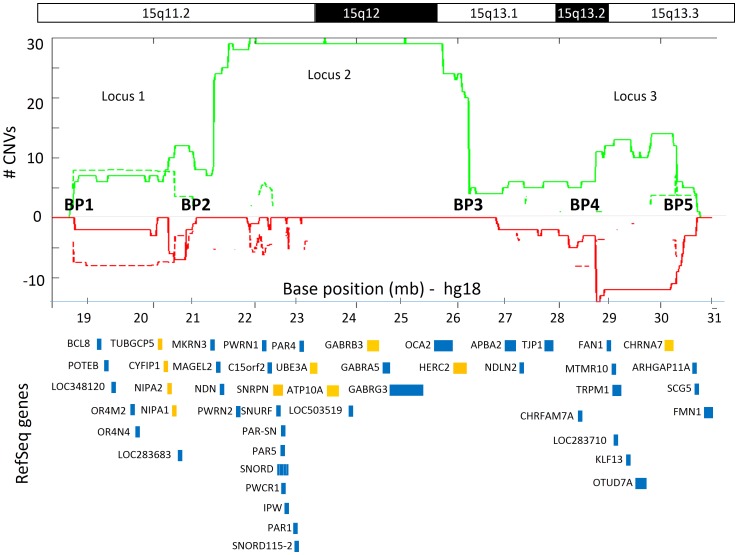
ASD susceptibility CNV loci on human chromosome 15 The number of individuals with duplications (green) and deletions (red) are plotted for both ASD cases (continuous lines) and controls (broken lines, on a log_2_ scale) along human chromosome 15q11.2-13.3. RefSeq genes overlapping with these regions are depicted in blue rectangles. Genes that have been associated with ASD according to AutDB [18] are colored in orange. The variation in CNV burden along the 15q11.2 – 13.3 region, suggests three distinct ASD susceptibility loci: Locus 1 within breakpoints (BP) 1-2, Locus 2 within BPs 2-3, and Locus 3 within BPs 4-5.

The next top-ranked CNV loci were located in three regions on human chromosome 15q11.2-13.3 (Figure 4). The largest of the three loci (15q11.2-q13.1) spanned 4.95 Mb between chromosomal breakpoints BP2 and BP3 and consisted predominantly of duplications among ASD subjects (31 duplications vs. 2 deletions). Two additional susceptibility loci on chromosome 15 (15q11.2 and 15q13.2-q13.3, within BP1-BP2 and BP4-BP5, respectively) were approximately half the size (∼2.4 Mb) and demonstrated equivalent prevalence of duplications and deletions in ASD individuals. A total of 44 RefSeq genes reside within these three regions, 10 of which have already been associated with ASD: *CYFIP1, NIPA1, NIPA2*, and *TUBGCP5* within the 15q11.2 locus; *UBE3A, ATP10A, GABRB3, SNRPN,* and *HERC2* within the 15q11.2-q13.1 locus; and *CHRNA7* within the 15q13.2-q13.3 locus. A closer inspection of the inheritance patterns of CNVs in these three genomic regions revealed that duplications tended to be *de novo* in origin, whereas deletions tended to be inherited (Table 2).

**Figure 5:**
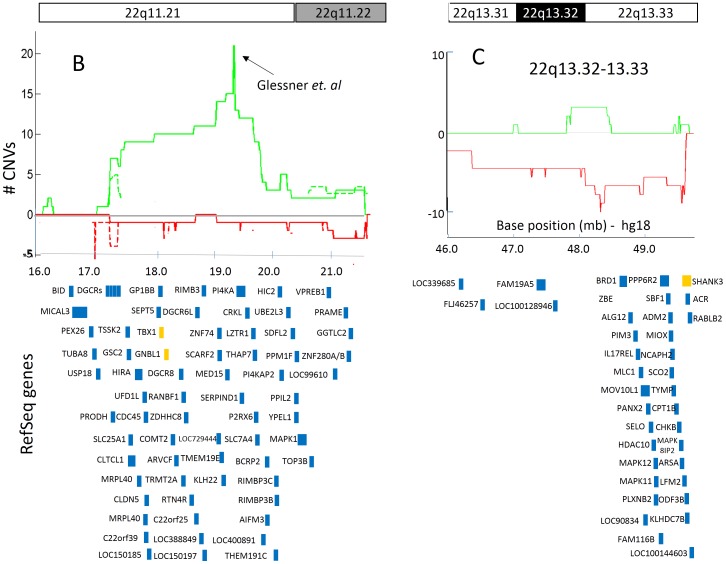
ASD susceptibility CNV loci on human chromosome 22. The number of individuals with duplications (green) and deletions (red) are plotted for both ASD cases (continuous lines) and controls (broken lines, on a log_2_ scale) along human chromosome 22q11.21-13.33. RefSeq genes overlapping with these regions are depicted in blue rectangles. Genes that have been associated with ASD according to AutDB [18] are colored in orange. (**A**) The CNV locus on 22q11.21 contains primarily copy number gains (duplications). A black arrow indicates the peak in duplications count due to the CNV data from Glessner *et. al*. (**B**) The CNV locus on 22q13.32-13.33 contains primarily copy number losses (deletions).

Two multigenic ASD susceptibility CNV loci were identified on human chromosome 22 (Figure 5). The 22q11.21 and 22q13.32-q13.33 loci spanned 2.5 Mb and 1.5 Mb, and overlapped with 51 and 33 genes, respectively. The CNV locus on 22q11.21 was predominantly enriched in duplications in ASD cases (21 duplications vs. 1 deletion), with a sharp increase in the number of duplications within a 20 kb region (chr22:19,345,000-19,365,000) that was implicated as a CNV enriched region in ASD cases (Glessner et al) (Figure 5A). Of the 51 genes within the 22q11.21 locus, two (*TBX1* and *GNBL1*) have already been associated with ASD. The locus on 22q13.32-q13.33 was primarily enriched in deletions among individuals with ASD (nine deletions vs. three duplications), and did not include CNVs in controls (Figure 5B). No previously characterized ASD-associated genes resided within the boundaries of this genomic locus; however, the ASD-associated gene SHANK3 is directly adjacent to the telomeric end of this region.

Finally, we examined the overlap of these eleven ASD susceptibility loci with CNV data from 4400 control individuals. Consequently, three of these loci (3q26.31, 4q35.2; Figure S3, and 15q11.2; Figure 4) showed a complete overlap with CNVs among controls, suggesting potential false positives, or polymorphic loci that confer lesser risk than initially estimated. In addition, three other ASD susceptibility loci (15q11.2-q13.1, 15q13.2-q13.3, and 22q11.21; Figures 4 and 5) demonstrated partial overlap with CNVs among controls, allowing us to refine the boundaries of this susceptibility loci.

## Discussion

The major goals of this study were to: (A) prioritize ASD susceptibility loci based on their CNV burden among ASD individuals, and (B) determine the genomic and genetic characteristics of these loci. The CNV dataset used for our analysis was curated from published scientific reports and primarily included CNVs that have been exclusively seen among individual with ASD in each of these studies. Yet, we compared these data with a control CNV dataset derived from three genome-wide CNV reports [19]–[21] to further refine the boundaries of our susceptibility loci and identify potential false-positives or lower-risk susceptibility loci. In addition, we contrasted our results with those of two other similar studies [28], [29], and a comprehensive review of the scientific literature describing ASD-associated CNVs [30]. We found that five (45.45%) of the susceptibility loci reported in this study were also identified in all of these other reports, and two other loci (18.2%) overlapped with one large population study of CNVs in human genetic disease [28] (Table S2). These overlapping findings support the validity of our analysis, especially given the relatively low frequency and widespread genomic distribution of ASD susceptibility CNV loci, as well as the differences in population makeup in the other studies.

The highest CNV burden in our study was seen in the 16p11.2 locus. This genomic locus have long been known as, a genetic risk factor for ASD [31], as well as other disorders including schizophrenia [32], developmental delay and cognitive impairment [33], major depressive disorder [34], and obesity [35]. Three genes within this locus (*SEZ6L2*, *MAPK3,* and *KCTD13)* have been independently identified as ASD genetic risk factors [25]–[27], but the genetic mechanisms by which deletions or duplications within the 16p11.2 locus contribute to ASD susceptibility remain unknown. Initial hints for potential functional mechanism might be found in a recent study in zebrafish [27] demonstrated that over-expression of the ASD-associated gene, *KCTD13,* led to decreased proliferation of neural progenitor cells and reduced head size, mirroring the microcephaly phenotype commonly seen in cases with 16p11.2 duplication. Alternatively, suppression of *KCTD13* led to increased neural progenitor cell proliferation and increased head size, mirroring the macrocephaly phenotype observed in many cases with 16p11.2 deletion. Likewise, another gene within the 16p11.2 locus, *TAOK2*, was recently shown to influence the formation of basal dendrites in the developing cortex [36]. Therefore, it is likely that these two genes, as well as other genes within the 16p11.2 susceptibility loci, act in concert and contribute to ASD susceptibility.

Our analysis implicated three distinct ASD loci within the 15q11-q13 genomic locus. Duplications within the 15q11.2-q13.1 region, located between chromosomal breakpoints (BPs) BP2-BP3, have long been strongly implicated in ASD pathogenesis [37], whereas deletions of this region are a primary cause of Angelman and Prader-Willi syndromes [38]. The 15q11.2-q13.1 region is flanked by two genomic loci, 15q11.2 and 15q13.2-q13.3 that have demonstrated association with not only autism but also other neurodevelopmental and neuropsychiatric disorders. CNVs within the 15q11.2 genomic loci (BP1-BP2) have been shown to confer risk to epilepsy and developmental delay [39], [40], while deletions of the 15q13 region (BP4-BP5) were associated with increased risk for intellectual disability and epilepsy [41]. Notably, the complete overlap of CNVs among controls with the 15q11.2 locus, as seen in other two others CNV loci in this study (3q26.31, and 4q35.2) support the two-hit premise of ASD etiology [42].

Four monogenic genomic loci were identified in our analysis that merit further investigation. The 9p24.3 locus, which contains the *DOCK8* gene overlaps with linkage regions identified in large autism extended pedigrees [43], [44], and disruption of *DOCK8* has previously been implicated in two cases of intellectual disability and developmental delay [45]. Therefore, while there is no direct evidence implicating *DOCK8* in ASD, these previous results and our findings argue for a potentially critical role for this gene in ASD susceptibility. The *BCL9* gene resides within a ∼1.5–Mb genomic region of the 1q21.1 locus in which both deletions and duplications can result in syndromes associated with numerous phenotypes, including autism [46]. Furthermore, common variants in the *BCL9* gene are associated with schizophrenia, bipolar disorder, and major depressive disorder [47]. *BCL9* is a component of the canonical Wnt signaling pathway, which has been proposed to be affected in ASD [48]. While there is no direct genetic evidence demonstrating that the *BCL9* gene confers genetic risk to ASD susceptibility, our analysis in combination with previous findings strongly implies a potential pathogenic role for this gene. Duplications of the 3q26.31 locus within the *NLGN1* gene were also seen among control individuals, thus questioning the association of this locus with ASD susceptibility [10]. Yet, the functional relevance of *NLGN1* to ASD is supported by a recent report identified a duplication of the *NLGN1* gene in an autistic patient with mild intellectual disability [49], as well as by functional studies demonstrating a role for this gene in neurite outgrowth via interactions with the ASD-associated gene NRXN1 [50]. Finally, the *KPNA3* gene at the 13q14.3 locus, has also been implicated as a potential schizophrenia susceptibility gene [51]. Further investigation will be required to ascertain the relevance of *KPNA3* to these two related psychiatric disorders.

The incorporation of CNV data from both large-scale whole-genome studies and smaller-scale case studies as a framework for ASD-related CNV loci characterization is the major strength of our study. However, this approach has some limitations. First, some of the studies in our dataset included genome-wide CNV data, while other studies focused on the identification of CNVs within specific genomic loci or with a specific mechanism of CNV inheritance. To account for the potential bias arising from this variation, we assigned different weights to studies based on the CNVs/Loci ratio reported in them, and incorporated this weight in the calculation of the CNV burden score (See methods). Another potential source for variation in our data is the differing CNV detection technologies used by the different studies. Accordingly, one might suspect that the higher burden scores observed among larger genomic regions (>500 kb) would be due to the greater likelihood of CNVs within these regions to be detected by all CNV detection methods, whereas smaller CNVs would not be detected by many of the older lower-resolution microarray platforms. While we cannot rule out this possibility, Sanders et. al [14], which employed a high-resolution CNV detection method, reported that the burden of CNVs in their study was remarkably similar to previously published results using lower-resolution CNV detection platforms.

The results of this analysis could have broad clinical and scientific implications. For example, one could use these loci as a guideline for the evaluation of chromosomal microarray (CMA) screening, a procedure that is being increasingly used in genetic evaluation of ASD subjects. Alternatively, the detailed characteristics provided for each of the CNV loci highlighted in the study may be used for further, in-depth exploration of the biological mechanism underlying their role in ASD susceptibility. An intriguing premise is whether the different genetic mechanisms trigger ASD susceptibility in subjects containing CNVs at distinct genomic loci, and whether these genetically diverse individuals present different ASD related phenotypes. We anticipate to increase the resolution of these analyses with continued updates to AutDB and additional control CNV data, which will subsequently provide both scientists and clinicians with a valuable resource for genetics research and clinical diagnostic efforts of ASD.

## Supporting Information

Figure S1 Distribution of CNV loci reports. Distribution of ‘major' and ‘minor' reports across CNV loci in AutDB[18], (A) Venn Diagram, (B) cumulative distribution function (cdf). (C) Top 1% reported CNV loci.
(TIF)

Figure S2 Distribution of CNV sizes. (A) Histogram of the log10 (CNV size) indicate that CNV sizes in our data have a lognormal distribution with a mean  =  42.8 kb. (B) CDF plots for the sizes of copy number gains (green), and copy number losses (red).
(TIF)

Figure S3 ASD susceptibility CNV loci on human chromosomes 3 & 4. The number of individuals with duplications (green) and deletions (red) are plotted for both ASD cases (continuous lines) and controls (broken lines) along human chromosomes 3q26.31 & 4q35.2. RefSeq genes overlapping with these regions are depicted in blue rectangles. Genes that have been associated with ASD according to AutDB [18] are colored in orange.
(TIF)

Table S1 Scientific reports of CNVs in ASD individuals. Details of the 48 scientific reports and their CNV data used in this study are listed in the table.
(XLS)

Table S2 CNV loci associated with ASD across different studies. A list of CNV loci associated with ASD from four different studies are depicted in the table. CNV loci highlighted in multiple studies are highlighted in bold.
(DOCX)
