# Supplementary figures and images for: Prioritization of Copy Number Variation Loci Associated with Autism from AutDB–An Integrative Multi-Study Genetic Database
Source: PLoS One. 2013 Jun 18;8(6):e66707. doi: 10.1371/journal.pone.0066707 (PMC3688962; doi:10.1371/journal.pone.0066707)

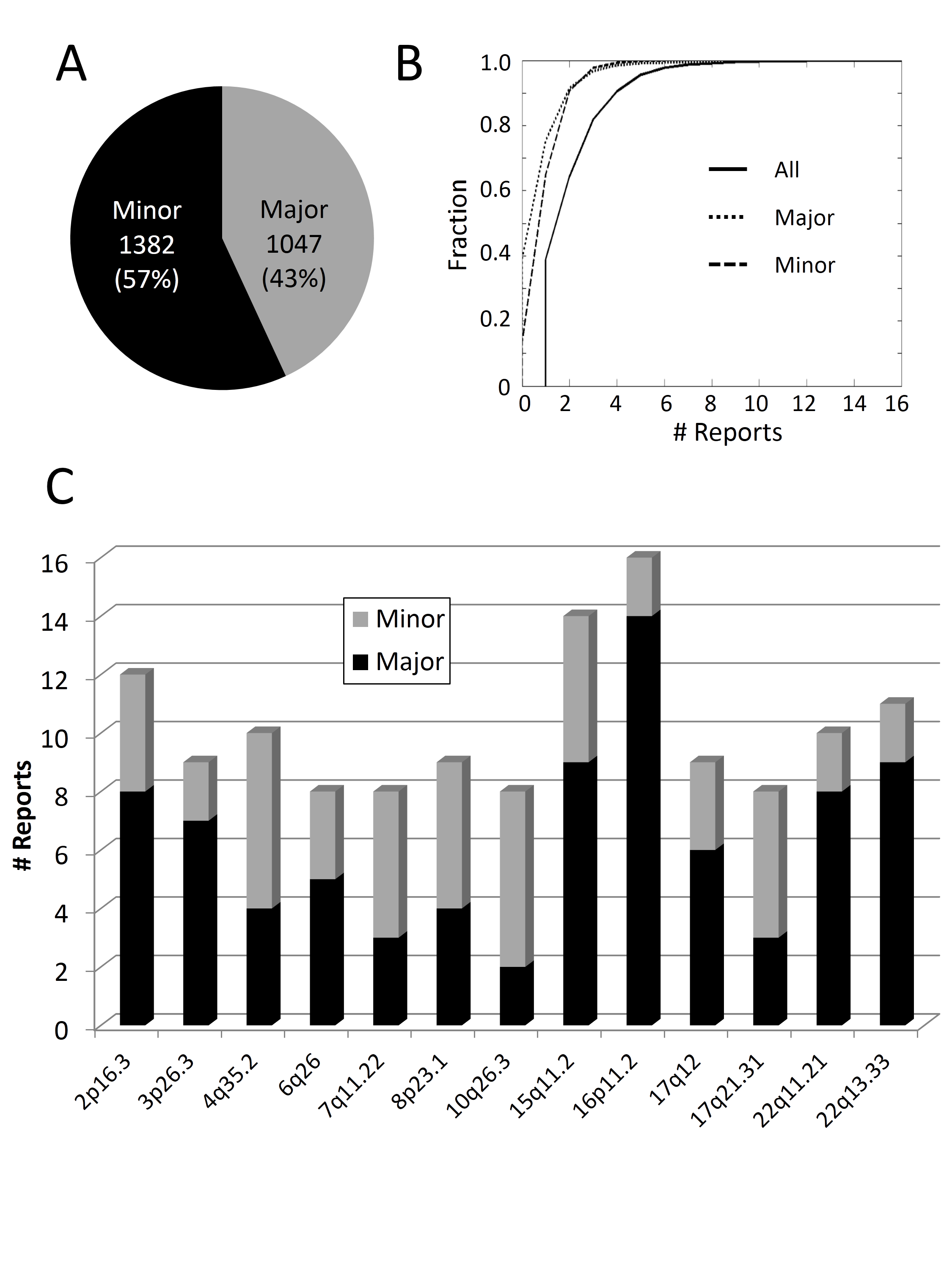

Supplement: Figure S1 — Distribution of CNV loci reports. Distribution of ‘major' and ‘minor' reports across CNV loci in AutDB[18], (A) Venn Diagram, (B) cumulative distribution function (cdf). (C) Top 1% reported CNV loci. (TIF) [file pone.0066707.s001.tif]

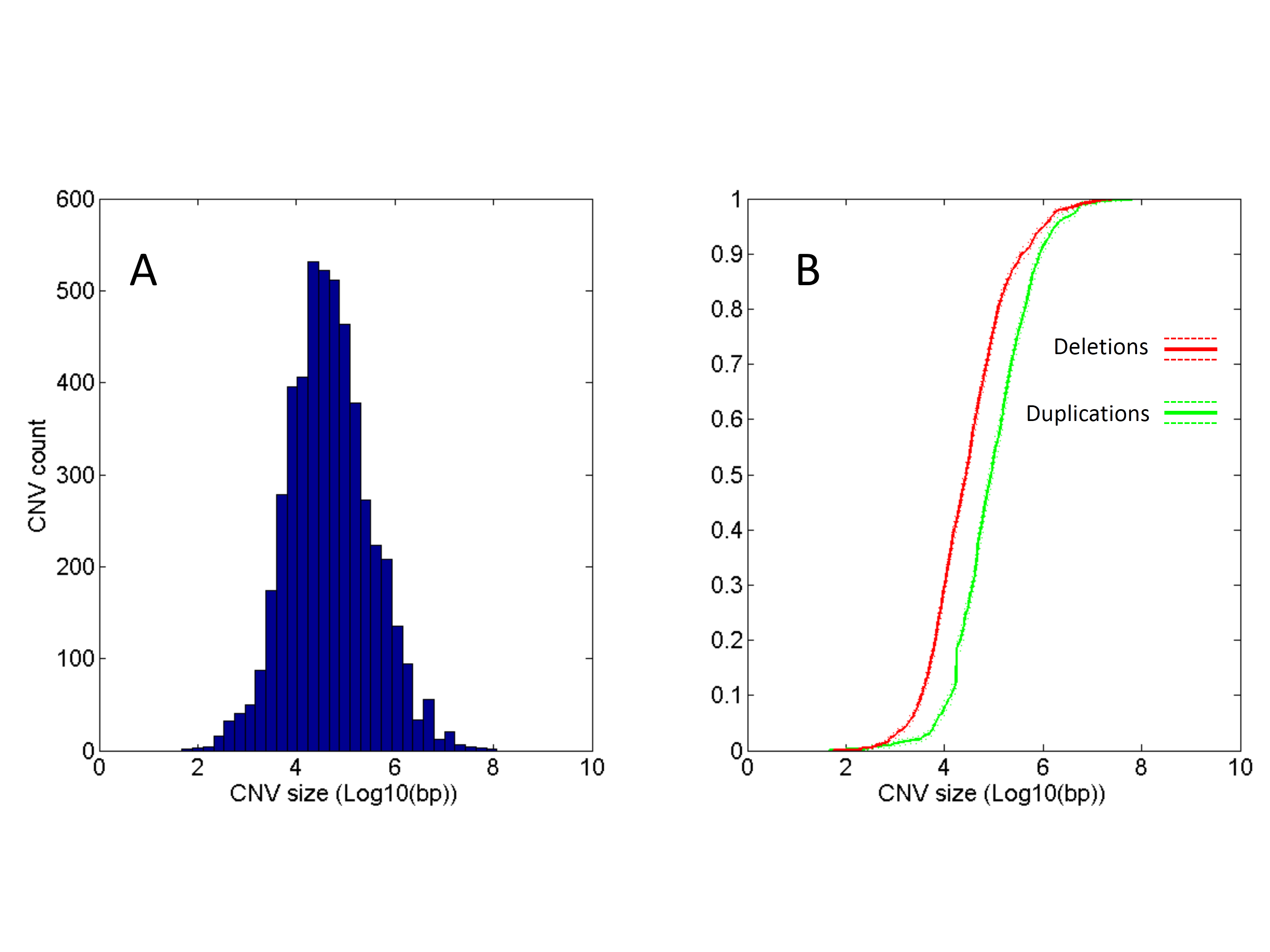

Supplement: Figure S2 — Distribution of CNV sizes. (A) Histogram of the log10 (CNV size) indicate that CNV sizes in our data have a lognormal distribution with a mean = 42.8 kb. (B) CDF plots for the sizes of copy number gains (green), and copy number losses (red). (TIF) [file pone.0066707.s002.tif]

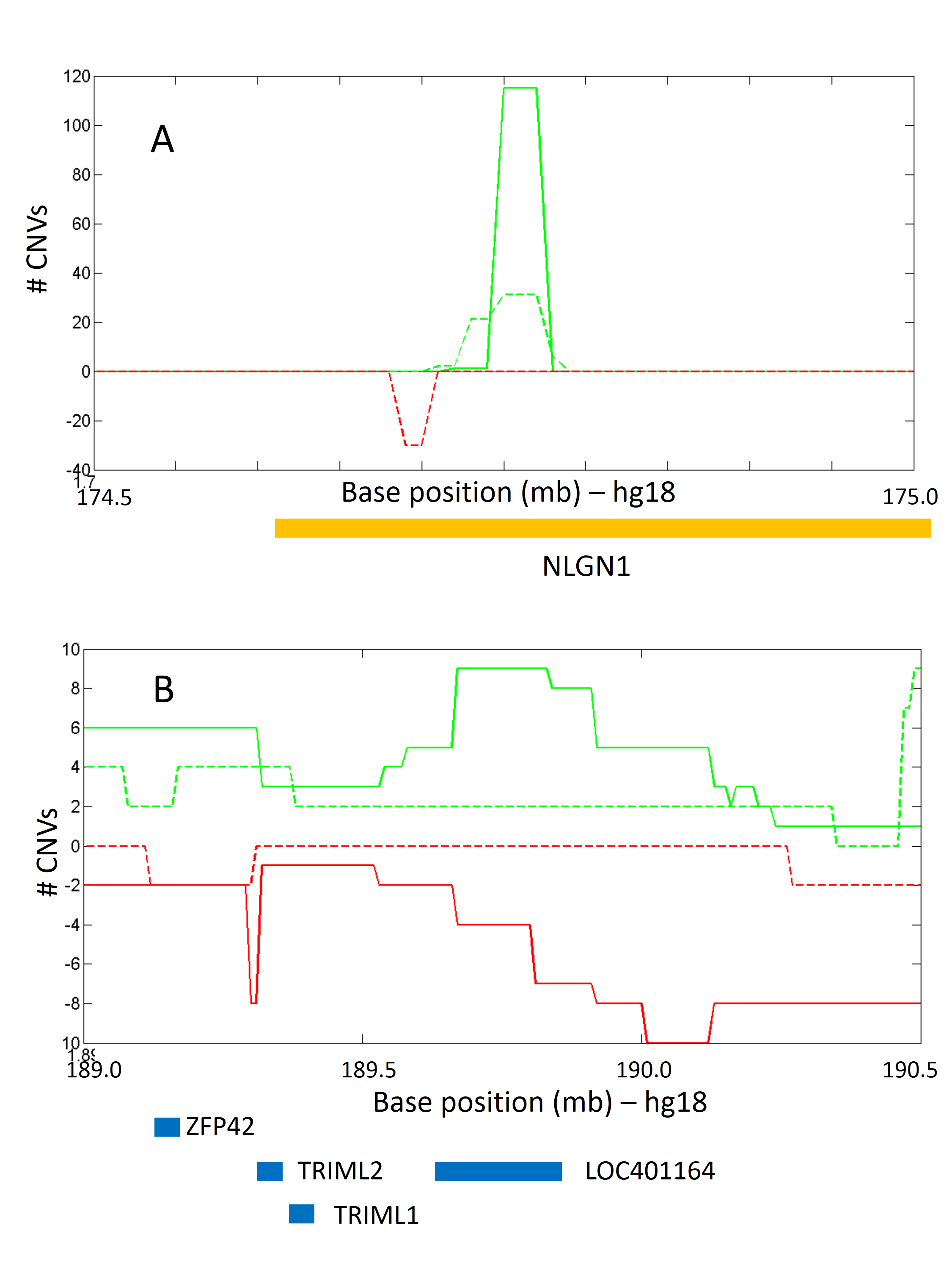

Supplement: Figure S3 — ASD susceptibility CNV loci on human chromosomes 3 & 4. The number of individuals with duplications (green) and deletions (red) are plotted for both ASD cases (continuous lines) and controls (broken lines) along human chromosomes 3q26.31 & 4q35.2. RefSeq genes overlapping with these regions are depicted in blue rectangles. Genes that have been associated with ASD according to AutDB [18] are colored in orange. (TIF) [file pone.0066707.s003.tif]
