# Supplementary material for: Prioritization of Copy Number Variation Loci Associated with Autism from AutDB–An Integrative Multi-Study Genetic Database
Source: PLoS One. 2013 Jun 18;8(6):e66707. doi: 10.1371/journal.pone.0066707 (PMC3688962; doi:10.1371/journal.pone.0066707)
Supplement: Table S2 — CNV loci associated with ASD across different studies. A list of CNV loci associated with ASD from four different studies are depicted in the table. CNV loci highlighted in multiple studies are highlighted in bold. (DOCX) [file pone.0066707.s005.docx]

**Table S2: CNV loci associated with ASD across different studies.**

| **Locus** | **AutDB** | **Marshal^1^** | **Kaminsky^2^** | **Itsara^3^** | **Total** |
| --- | --- | --- | --- | --- | --- |
| **1q21.1** | **X** | **X** | **X** | **X** | **4** |
| 1q44 |  | X |  |  | 1 |
| 2p16.3 |  | X |  |  | 1 |
| 2q32.1 |  |  |  | X | 1 |
| 2q37.3 |  |  |  | X | 1 |
| 3q26.3 | X |  |  |  | 1 |
| **3q29** |  |  | **X** | **X** | **2** |
| 4q35.2 | X |  |  |  | 1 |
| 5p15.2 |  | X |  |  | 1 |
| 5p15.3 |  | X |  |  | 1 |
| 5q35 |  |  | X |  | 1 |
| 6q25.2-q27 |  | X |  |  | 1 |
| 7q11.22 |  | X |  |  | 1 |
| 7q11.23 |  |  | X |  | 1 |
| 7q35-q36 |  | X |  |  | 1 |
| 7q36.1 |  | X |  |  | 1 |
| 7q36.2 |  | X |  |  | 1 |
| 8p22 |  | X |  |  | 1 |
| 8p23.1 |  |  | X |  | 1 |
| 9p24.3 | X |  |  |  | 1 |
| 9q33.1 |  | X |  |  | 1 |
| 11q14.1 |  |  |  | X | 1 |
| 13q14.3 | X |  |  |  | 1 |
| **15q11.2** | **X** |  |  | **X** | **2** |
| **15q11.2-12** | **X** | **X** | **X** | **X** | **4** |
| **15q13.1-13.3** | **X** | **X** | **X** | **X** | **4** |
| 15q21.1 |  | X |  |  | 1 |
| 15q25 |  |  |  | X | 1 |
| **16p11.2** | **X** | **X** | **X** | **X** | **4** |
| 16p12 |  |  |  | X | 1 |
| 16p13.11 |  |  | X |  | 1 |
| 16p13.3 |  | X |  |  | 1 |
| 16q21 |  |  |  | X | 1 |
| 16q23.3 |  |  |  | X | 1 |
| 17p11.2 |  |  | X |  | 1 |
| 17p12 |  |  |  | X | 1 |
| 17q12 |  |  | X |  | 1 |
| 17q21.31 |  |  | X |  | 1 |
| 19p12 |  | X |  |  | 1 |
| 20p12 |  | X |  |  | 1 |
| **22q11.21** | **X** | **X** | **X** | **X** | **4** |
| 22q12.3 |  | X |  |  | 1 |
| **22q13.32-13.33** | **X** |  |  | **X** | **2** |
| Xp22.11 |  | X |  |  | 1 |
| **Total** | **15** | **22** | **13** | **16** |  |

CNV loci highlighted in multiple studies are highlighted in bold.

1. Marshall, C.R., and Scherer, S.W. (2012). Detection and characterization of copy number variation in autism spectrum disorder. Methods in molecular biology (Clifton, NJ 838, 115-135.

2. Kaminsky, E.B., Kaul, V., Paschall, J., Church, D.M., Bunke, B., Kunig, D., Moreno-De-Luca, D., Moreno-De-Luca, A., Mulle, J.G., Warren, S.T., et al. (2011). An evidence-based approach to establish the functional and clinical significance of copy number variants in intellectual and developmental disabilities. Genet Med 13, 777-784.

3. Itsara, A., Cooper, G.M., Baker, C., Girirajan, S., Li, J., Absher, D., Krauss, R.M., Myers, R.M., Ridker, P.M., Chasman, D.I., et al. (2009). Population analysis of large copy number variants and hotspots of human genetic disease. Am J Hum Genet 84, 148-161.
